# Skin Barrier Function in Infants: Update and Outlook

**DOI:** 10.3390/pharmaceutics14020433

**Published:** 2022-02-17

**Authors:** Annisa Rahma, Majella E. Lane

**Affiliations:** 1 Pharmaceutics Department, School of Pharmacy, Institut Teknologi Bandung, Bandung 40132, Indonesia; 2 School of Pharmacy, University College London, 29-39 Brunswick Square, London WC1N 1AX, UK; m.lane@ucl.ac.uk

**Keywords:** atopic dermatitis, baby, diaper dermatitis, paediatric, permeability, skin care, wipes

## Abstract

A good understanding of infant skin should provide a rationale for optimum management of the health of this integument. In this review, we discuss the skin barrier function of infants, particularly with reference to the use of diapers and baby wipes. The skin barrier of newborns continues to develop with age. Two years after birth, the barrier properties of infant skin closely resemble those of adult skin. However, several risk factors may contribute to impaired skin barrier and altered skin permeability in infants. Problems may arise from the use of diapers and baby wipes. The skin covered by a diaper is effectively an occluded environment, and thus is vulnerable to over-hydration. To date there has been no published information regarding dermal absorption of ingredients contained in baby wipes. Similarly, dermal absorption of topical ingredients in infants with underlying skin conditions has not been widely explored. Clearly, there are serious ethical concerns related to conducting skin permeation studies on infant skin. However, the increasing availability of non-invasive methods for in vivo studies is encouraging and offers new directions for studying this important patient group.

## 1. Introduction

The aim of this review is to examine what is known about the skin barrier function in infants, with a primary focus on the infant population where there is regular use of diapers and cleansing wipes. The effects of cleansing wipes on the barrier function of the skin sites where diapers are worn are reviewed in detail. Furthermore, safety concerns concerning repeated use of cleansing wipes for the diaper area are also discussed. For the sake of brevity, disposable cleansing wipes intended to cleanse the infant diaper are referred to as baby wipes in this analysis.

To our knowledge, this topic has not been examined in detail previously. Therefore, we set out in Table 1 the nomenclature to clarify the age groups of children that are the focus of this review. Although the skin barrier function of neonates and infants has been reviewed [1], it is important to note that the skin physiology and function in preterm infants is less developed compared with full-term infants [2,3,4,5,6,7]. A number of studies have also confirmed a high incidence of percutaneous toxicity from topical substances in preterm infants [3,8,9,10,11]. 

The skin barrier function in humans is primarily attributed to the stratum corneum (SC). The SC functions to protect the body from the external environment and to maintain sufficient hydration by regulating the rate of water loss [1,6,11,12]. The physiology of the skin develops gradually during the gestation period. At birth, full-term newborns have a well-defined SC, although gradual maturation continues to take place over time, eventually developing into competent adult skin [6,11,12].

From an anatomical point of view, the differences between infant skin and adult skin are limited. The most distinctive characteristics of infant skin are a thinner SC, thinner epidermal layer, and smaller corneocytes [2]. In terms of skin physiology, infants have a less mature barrier function. Firstly, the loss of water vapor through the skin, as will be discussed later, is significantly higher compared with adults [13]. Secondly, the “acid mantle” of the skin surface is not yet formed [14]. The term “acid mantle” was first described by Schade and Marchionini in 1928, and describes the pH of the skin surface as measured using an electrometric technique [15]. The acid mantle of the skin surface is thought to be formed from filaggrin degradation products [16] and acidic excretion products from sebaceous and sweat glands [17,18]. The role of skin pH in maintaining skin barrier function, as will be discussed later, remains to be fully understood. However, the term “skin pH” itself is controversial, since pH is a concept that only has meaning in an aqueous environment.

A further problem in infants is the use of diapers and baby wipes that may also contribute to an impaired skin barrier. In infants, the diaper area is occluded, and this area is managed by the use of products that contain surfactants. In vitro human studies have shown that, depending on the circumstances, surfactants may damage the SC by interacting with lipids [19]. Consequently, topical products intended for infant skin should be carefully formulated. These preparations should minimize the use of irritating/sensitizing ingredients and should also be free from microbial contamination [19]. 

## 2. Skin Structure and Function

**Figure 1:**
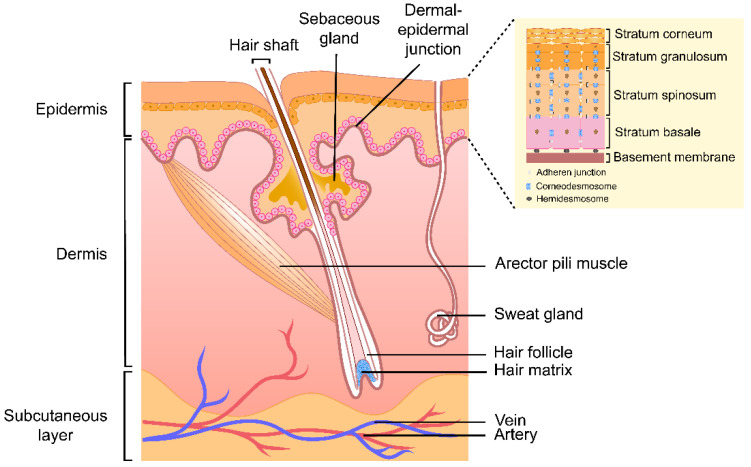
Anatomy of normal human skin.

Skin serves as a protective barrier for the human body from external factors, such as UV radiation, pathogens, and harmful chemicals. This organ also contributes to skin temperature regulation, hydration and sensory perception [20]. In order to fulfil these functions, the skin is constructed of multiple layers that are morphologically distinctive (Figure 1). Although this section reviews what is generally known about adult skin structure, the skin barrier function of infants develops to resemble that of adults. 

The upper layer of the skin is a highly stratified layer known as the epidermis. The epidermis is composed of five layers from the outermost to the bottom: the SC, stratum lucidum, stratum granulosum (granular layer), stratum spinosum (spinous layer) and stratum basale (basal layer) [20]. The last four layers constitute the viable epidermis [21]. The major cellular components of the epidermis layer, keratinocytes, gradually differentiate and migrate outwards from the stratum basale to the SC [20]. Upon complete maturation, keratinocytes are transformed into corneocytes. Underneath the epidermis layer is the dermis layer, which is composed of connective tissue where the skin appendages originate; these are the hair follicles, sebaceous glands, and sweat glands [1,6,11,12]. The epidermis and dermis layers are connected and mechanically supported by the dermal–epidermal junction (basement membrane). 

### 2.1. Epidermis

**Figure 2:**
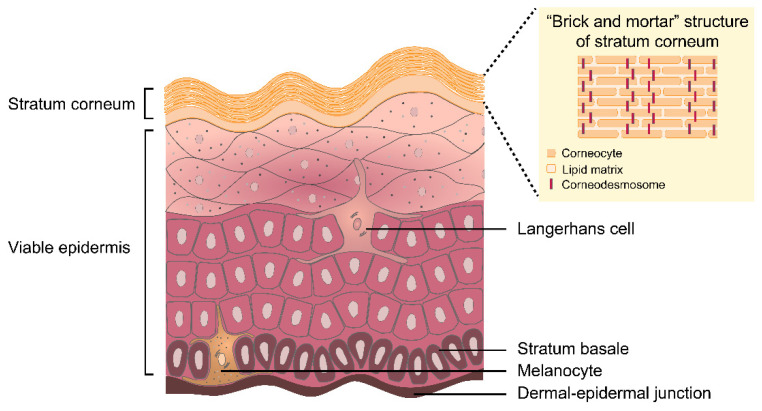
The location of dendritic cells (Langerhans cells and melanocytes) in the viable epidermis.

The epidermis is composed of the SC and the viable epidermis (Figure 2). As described earlier, the main role of the SC is to provide a physical barrier. The SC has been reported to contain approximately 15% water, 15% lipids, and 70% proteins [22]. The structure of the SC resembles a ‘brick and mortar’ wall, in which the corneocytes as the ‘bricks’ are interconnected by the ‘mortar’ of a lipid matrix [23]. The lipid matrix, also described as intercellular lipids, is a highly ordered phase consisting of ceramides, cholesterol, and fatty acids [23]. The corneocytes are individually covered by a cell envelope and covalently-bonded lipids [22,24]. The corneocytes are attached to each other by corneodesmosomes [23,25]. The corneodesmosomes are involved in the renewal of the SC, termed desquamation, where the corneodesmosomes are degraded as corneocyte maturation takes place [23,25].

While the corneocytes and corneodesmosomes provide the physical resistance of the SC, the intercellular lipids are responsible for other functional characteristics, including water diffusion/transport and permeability [22]. The viable epidermis is responsible for replenishing the cells of the SC layer and regulating the immune response against potentially harmful external threats [20]. In the lowest layer of the viable epidermis (basal layer), the epidermal stem cells begin to differentiate into keratinocytes, undergo further maturation, and move to the outermost surface of the skin [20]. In normal human skin, this process requires roughly 28 days to complete [20]. In the first stage of keratinocyte differentiation, the loss of cell nuclei and the formation of the cornified cell envelope occurs in the granular layer [26]. At the same time, filaggrin undergoes enzymatic degradation to produce natural moisturizing factor (NMF) [27]. NMF is composed of amino acids, organic acids, sugars, and other low molecular weight hydrophilic compounds [28]. These compounds play an important role in maintaining skin hydration [28]. The keratinocytes are flattened and transformed into rigid corneocytes as they progress to the outer layers of the skin [26]. The intercellular lipid matrix of the SC originates from a mixture of lipids (ceramides, cholesterol, and fatty acids) secreted by the lamellar bodies located in the spinous layer. During the cornification of the keratinocytes, this lipid mixture localizes to fill in the intercellular spaces between the epidermal cells, finally forming the ‘mortar’ of the SC [21,26]. 

Two types of skin-resident dendritic cells, namely Langerhans cells (LC) and melanocytes, are located in the viable epidermis. The epidermal LC are antigen-presenting cells, and the cells process antigens, migrate to the regional lymph nodes, and present the antigen fragments to the T cells for further immune responses [20]. The melanocytes reside in the basal layer. These cells produce melanin, which contributes to skin pigmentation [21]. Skin pigmentation is a defense mechanism against UV radiation. In normal skin, UV radiation induces the synthesis of melanin. The melanin is then transferred to the keratinocytes in order to prevent DNA damage [21]. 

### 2.2. Dermal-Epidermal Junction

The dermal-epidermal junction, also known as the basement membrane, acts as the barrier between the epidermis to the dermis but also mediates various functional interactions between these two layers [20]. Among the interactions are migration of LC and melanocytes as well as signal transduction between the extracellular matrix (ECM) and the keratinocytes. These activities are involved in the regulation of cell adhesion, proliferation, and differentiation [29]. The upper part of the dermal-epidermal junction, the basal plasma membrane of keratinocytes, contains hemidesmosomes [29]. Hemidesmosomes are multiprotein complexes that provide adhesion between the epidermal and dermal layer, hence they also contribute to the structural integrity of the skin [20].

### 2.3. Dermis

The dermis is located between the dermal-epidermal junction and the subcutaneous layer. The dermis provides nourishment and mechanical support to the skin and the appendages [20]. The biomechanical characteristics of the dermis are a result of the major constituent—the ECM, that has favorable tensile strength and viscoelastic properties. The ECM is comprised of fibroblast cells and related secretion products (collagen, elastin, proteoglycans, glycosaminoglycans, and glycoproteins). Collagen and elastin are responsible for the physical resilience of the skin while the proteoglycans are known to possess high water retention capacity [20]. Glycosaminoglycans and glycoproteins are ECM components that play an important role in cell adhesion [20].

## 3. Infant Skin Maturation

The skin development process starts during early gestation—in the first trimester [2]. Structural and functional maturation continues until the full-term, and the SC is fully formed at 34-weeks of gestational age [2]. In the case of preterm newborns, the skin function is not comparable to full-term newborns at least until 2–3 weeks after birth [30]. At birth, newborn skin is covered by the *vernix caseosa,* a protective coating composed of water, lipids, proteins, and shed corneocytes [2]. The *vernix caseosa* provides epidermal barrier function to newborns [31,32]. However, the mechanisms underlying this are yet to be understood. It has been suggested that the lipids contained in the *vernix caseosa* act as a hydrophobic barrier which can regulate transepidermal water loss (TEWL) in newborn skin [33]. The *vernix caseosa* is removed during the first washing of newborns, causing newborn skin to be drier and more vulnerable compared with fetal skin during the gestation period [34]. Moreover, important regulatory processes of newborn skin, as will be discussed in a later section, are not yet functional [27]. Therefore, the World Health Organization (WHO) recommends that newborns should not be bathed in the first six hours after birth [35]. 

Infant skin maturation continues after birth, particularly during the first year of life [2]. The structural, compositional and functional differences between infant skin and adult skin are summarized in Table 2. In the second year, infant skin more closely resembles adult skin [2]. 

### 3.1. Structure and Composition

The epidermis in full-term infants is well-formed and there are only minor structural differences between infant skin and adult skin [39]. However, in preterm neonates the skin barrier function is compromised compared with full-term newborns because of a remarkably thinner epidermis and SC [40]. Another distinct characteristic of infant skin is the organization of the dermis. At birth, the dermal ECM of full-term neonates contains less dense collagen fibril bundles compared with adult skin [2]. Although separate layers of papillary and reticular dermis are already formed, the collagen fibers in these two layers are not markedly different [2]. In terms of skin composition, newborn skin lacks water, NMF, and melanin content—which explains the physical appearance of newborn skin (drier and less pigmented) compared with adult skin [2,38].

### 3.2. Function

#### 3.2.1. Cell Turnover

As noted earlier, the skin turnover is driven by keratinocyte proliferation and maturation to corneocytes, followed by the desquamation process. Stamatas et al. (2010) performed an in vivo study regarding the microstructure and proliferation of epidermal cells in infants compared with adults [36]. Three different sites were examined, namely the inner arm, dorsal forearm, and lower thigh. In this work, the size of corneocytes was observed using a light microscope following SC collection using D-Squame tapes. In addition, the rate of keratinocyte proliferation was studied using fluorescence spectroscopy. A fiber optic probe was placed on the skin site and the excitation spectra from 240 to 320 nm were recorded. The fluorescence of tryptophan, with an excitation wavelength of 295 nm, was used as the marker of epidermal cell proliferation. The findings showed that the size of corneocytes in infants was significantly smaller compared with that of adults (*p* < 0.05) [36]. The rate of epidermal cell proliferation in newborns and infants aged up to 3 months was higher than that of older infants and adults (*p* < 0.05) [36]. It was suggested that the high proliferation rate lead to a smaller size of epidermal cells and higher cell density among infants compared with adults [36]. Two years after birth, the skin turnover rate was similar to adults (*p* > 0.05) [36].

In another study, Hoeger and Enzmann (2002) investigated the desquamation rate in infants at 3, 30, and 90 days after birth (*n* = 202) [41]. Polyacrylate-coated discs were pressed on the skin site (5 s contact) and subsequently removed. The opacity of the discs was measured by using image analysis software. The opacity per unit area represented the amount of corneocytes removed from the skin. The desquamation rate in infants was found to be site-specific. The facial area and the forearm demonstrated significantly higher desquamation rates 90 days after birth compared to 3 days after birth (*p* < 0.001). In contrast, desquamation in the buttock area at 90 days was significantly lower compared to results obtained at 3 days (*p* < 0.05) [41]. A number of studies have confirmed similar findings for adult skin [42,43]. A study by Roberts and Marks (1980) reported that desquamation rate, as determined using a scrub technique, was ranked as follows: forearm > back > thigh > upper arm > abdomen [43]. Mohammed et al. (2012) investigated corneocyte surface area for various skin sites by using tape stripping and immunostaining [42]. In this study, corneocyte surface area showed a linear correlation with corneocyte maturity [42]. Corneocyte surface area was ranked as follows: cheek > wrist > forearm > abdomen [42]. The authors suggested that the differences in corneocyte surface area for different skin sites may be related to environmental exposure [42]. For instance, larger corneocytes were found in the skin sites protected by clothing [42,44,45,46].

#### 3.2.2. Hydration and Water-holding Capacity

The water content of the SC, also referred to as the SC hydration, is essential for maintaining skin barrier function. SC hydration affects the maturation of epidermal and dermal cells, the desquamation process, and expression of keratins and proteins [4]. However, over-hydration of the SC may cause adverse effects such as perturbation of the SC lamellar structure [4]. The hydration of the SC is maintained by the presence of NMF [28]. 

Following removal of the *vernix caseosa,* the skin surface of newborns is rougher and dryer compared to older infants because of the low water content. The skin hydration improves over the next three months, reaching a hydration level that exceeds that of adult skin [41,47]. The skin hydration reaches the highest level between the age of 3 and 12 months [13]. This is considered to arise from functional maturation of the sweat glands [48]. Although infants have higher skin hydration compared to adults, their water-holding capacity is limited—characterized by rapid sorption-desorption of water molecules [2]. This appears to reflect the low concentration of NMF, composed of hydrophilic compounds that are essential for water retention capacity in the skin [28].

#### 3.2.3. Immunological Barrier

In infant skin, the LC which are responsible for T cell activation, are not fully mature [38]. However, bacterial colonization starts gradually with an evolving pattern, due to the specific features of infant skin (higher pH, low sebaceous activity, drier surface) [49]. Initially, the skin microbiome is dominated by firmicutes (mostly *Staphylococci*). As the skin maturation continues, actinobacteria, proteobacteria, and bacteroidetes begin to colonize the skin [50]. Bacterial colonization of infant skin is the origin of the innate immune response [51]. Finally, when the skin reaches full maturation, the typical microorganisms that reside on the skin are the firmicutes, actinobacteria, and phyla proteobacteria [50].

#### 3.2.4. pH

By definition, pH is the logarithm of hydrogen ion activity in an aqueous solution. Potentiometric instruments have been developed to measure the pH of the skin surface. Prior to measurement of skin pH, a planar electrode is wetted with water. The electrode is then placed onto the skin. Under these circumstances, water-soluble compounds that are present on the skin surface are dissolved in the water applied. The recorded pH value is therefore an apparent value measured at the interface between the skin surface and the electrode [52]. Another important consideration is that the SC also contains lipids. Therefore, the environment of the SC during skin pH measurement is not a simple aqueous solution [52]. For these reasons, the interpretation and meaning of skin pH measurements should be treated with caution.

In the intrauterine environment, fetal skin is covered by the *vernix caseosa.* The *vernix caseosa* and the amniotic fluid are reported to be mildly alkaline (pH > 7) [31,32,53]. It has been suggested that both the *vernix caseosa* and the amniotic fluid impart a neutral pH (6.6–7.5) to the newborn skin surface [14,34,47,54]. However, as noted, the skin pH remains a topic of considerable debate. It has been reported that skin acidification begins immediately after birth, reaching a pH range of 5–6 in the first few days of life [54], and continues to drop up to the first month [41]. One study investigated the change in skin pH in full-term newborns within 48 h after birth [54]. In the study, the pH values of various skin areas (forehead, upper back, abdomen, groin, flexor forearm, palms, and soles) were measured using a flat glass electrode pH meter. The findings showed that the mean skin pH values collected at 24 h were significantly higher (*p* < 0.05) compared to measurements at 48 h [54]. A similar study was conducted on infants at the following time points: 3 days, 4 weeks, and 12 weeks after birth [31]. In this study, the skin pH was measured on four different sites, namely forehead, cheek, volar forearm, and gluteal surface. It was reported that the skin pH at all skin sites decreased significantly (*p* < 0.01) from 3 days after birth to 90 days after birth. The greatest change was observed on the forehead, namely, −1.31 units of pH [31].

The accelerated skin acidification process is thought to be a result of the maturation of sebaceous glands and sweat glands, that allows the production of fatty acids, amino acids, and organic acids [4]. However, Behrendt and Green (1957) proposed that the pH of newborn skin might not reflect the pH of the SC [53]. It was proposed that the alkaline pH of newborn skin observed on the first day after birth might be associated with the presence of residual *vernix caseosa* on the skin surface [53,55]. One month after birth, the average skin pH is reportedly comparable to adult skin (pH 4–6) [54,56]. 

It has been proposed that the importance of skin acidification is related to the fact that the maturation and maintenance processes of skin barrier function are pH-sensitive [57,58,59,60]. Included in these pH-sensitive mechanisms are desquamation [57], immunological responses [59], and organization of intercellular lipids [17,58,60,61]. As discussed earlier, organization of SC lipids requires a specific ratio of ceramides, cholesterol, and fatty acids [62]. One of the enzymes required for SC lipid synthesis, epidermal β-glucocerebrosidase, demonstrated optimum activity at an acidic pH (5.6) in an in vitro enzyme assay [63]. In addition, Mauro et al. (1998) reported delayed maturation of intercellular lamellar bodies at neutral pH (pH = 7.4) [60]. Acetone-treated mouse skin (TEWL > 5mg/cm^2^/h) was exposed to buffer solutions with pH values of 5.5 or 7.4. The TEWL at 24 h was significantly higher in the group treated with the pH 7.4 buffer (*p* < 0.05) compared with the pH 5.5 buffer [60]. Microscopic examination of skin biopsies showed that the secreted lamellar bodies had delayed maturation for the pH 7.4 treatment compared with pH 5.5 [60]. It is important to note that this study was conducted on mice. Therefore, the findings may not necessarily be extrapolated to humans. However, it has been recognized that secretion and maturation of intercellular lamellar bodies is an important response to skin barrier disruption [61]. The lamellar body needs to mature before secretion of contents into the intercellular spaces of the SC [61]. 

#### 3.2.5. Photoprotection

The skin provides protection from harmful UV radiation through the synthesis of melanin. Infant skin reportedly has a lower melanin content compared with adult skin [64]. This was investigated using diffuse-reflectance spectroscopy [64]. This finding might reflect the state of melanocytes in infant skin, as they have not fully matured [38]. However, Moise et al. (1999) reported that infants spent less time outdoor and received lower UV exposure compared with children aged 2.5 years [65]. In the study, the impact of UV exposure on the skin was assessed by measuring erythema effective dose (EED) using polysulphone film badges on the shoulder and chest. The EED score was consistent with the time spent outdoor (exposure time). 

There is increasing concern that the risk of UV-induced skin damage is likely to be higher in infants [2,64,66]. This is because of the thinner epidermis and highly hydrated SC in infants, that potentially result in reduced light scattering with exposure to UV rays. Further studies are required to confirm whether infants are more susceptible to UV exposure-related skin damage compared with adults. The American Academy of Pediatrics recommends that the use of sunscreen as a preventive measure for infant skin protection should be restricted to areas that are not covered by clothes [66]. This is to minimize the skin absorption of sunscreen ingredients [66].

#### 3.2.6. Sebaceous Activity

Sebaceous glands excrete sebum, a mixture of lipids (squalene, wax esters, cholesterol esters, and triglycerides) that constitute the major skin surface lipids [67]. Therefore, measurement of sebum production can be performed based on the content of skin surface lipids. Two studies reported that full-term newborn skin has considerable skin surface lipid production, but the sebum level decreases drastically by 6 months [68,69]. A correlation between the skin surface lipid production in infants and mothers has been investigated [68]. In this study, a decrease in skin surface lipid production on the forehead of newborns and their mothers was observed within a week after birth, as measured using an absorbent filter paper method. The trends in skin lipid production in newborns and their mothers were found to be consistent. It was suggested that the rate of skin surface lipid production in newborns at birth is a result of maternal hormonal stimulation before birth [68,69]. A later study reported that the ratio of skin surface lipid compositions, as measured on the forehead, remained stable from 6 months after birth until 6 years [70].

#### 3.2.7. Transepidermal Water Loss (TEWL)

The structural integrity of the SC is generally characterized by TEWL. TEWL is defined as the flux of water diffusing through the SC. The TEWL value is expressed as vapor flux over a defined skin area per unit time. Currently, a range of TEWL measurement instruments has been developed. Open-chamber TEWL measuring instruments have been widely used [71]. This type of instrument consists of a probe with an open cylinder, where the upper end of the cylinder is open to the ambient atmosphere. To measure the TEWL, a probe is placed on the skin area. The probe measures the gradient of vapor pressure at a known distance above the skin relative to the skin surface [46]. This is interpreted as the evaporative water loss [46]. From a practical point of view, TEWL measurement using the open-chamber method has limitations, namely potential interference from environmental factors such as air current, temperature and humidity [46,71,72]. A modified open-chamber instrument has been developed (also known as the semi-open system) where the upper part of the chamber is equipped with a grid to minimize the interference from ambient air currents [71,72]. The closed-chamber system has been developed to prevent such interference from environmental factors during measurement. Since the chamber is closed, the measurement is based on accumulative water vapor instead of water vapor gradient [71,72]. There is a concern that the accumulation of water vapor can saturate the chamber and interfere with the measurement [73]. To address this issue, a novel closed-chamber instrument equipped with a condenser has been developed; the role of the condenser is to lower the temperature of the water vapour below the freezing point of water (−7 °C) [72,73].

In normal adult skin, the TEWL is reported to range from 6 to 8 g/m^2^/h. Higher TEWL values may reflect epidermal barrier dysfunction [21]. A number of studies have confirmed that the rate of TEWL is age- and site-dependent [5,44]. Hammarlund and Sedin (1979) measured TEWL values of preterm newborns and full-term newborns at various sites: the chest, interscapular area, and the buttock [5]. The average TEWL values from the three sites were found to be inversely proportional to the gestational age. Moreover, the average TEWL values of preterm newborns (up to 75 g/m^2^/h) were higher than for full-term newborns (6–8 g/m^2^/h) [21]. As a consequence, preterm newborns are likely to be at higher risk of heat loss through evaporation, particularly in low humidity environments (<50%) [5]. The high TEWL values in preterm newborns may reflect the thinner epidermis, extensive dermal capillary networks, and higher skin hydration compared with full-term infants [5,44].

Nikolovski et al. (2008) investigated TEWL values in infants aged 3 to 12 month old as compared to values in children and adults aged from 14 to 73 years [13]. TEWL measurement was conducted on the lower dorsal and upper ventral arms. The study reported that the TEWL values from both skin sites in healthy full-term infants fluctuated during the first year of life [13]. In addition, the TEWL values from lower dorsal and upper ventral arms in infants (*n* = 50) were reported to be significantly higher than in adults (*p* < 0.01) [13]. In another study, older infants (aged 8 to 24 months) exhibited TEWL values that were similar to adults, (*p* < 0.05), based on measurements of the volar forearm and buttock [14]. 

As discussed earlier, different instruments use varying approaches to measure TEWL. The open-chamber system can be affected by environmental factors (air current, temperature, and relative humidity) [46,71,72,73]. Therefore, care should be taken when making direct comparison of TEWL values collected with instruments of differing design. 

## 4. Percutaneous Absorption in Infants

**Figure 3:**
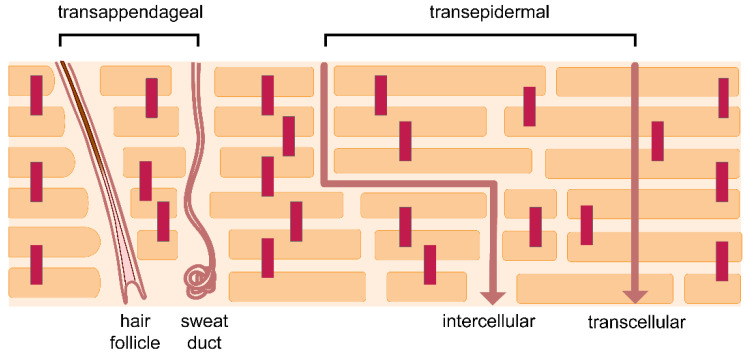
Skin permeation pathways.

Skin permeation pathways are illustrated in Figure 3. A molecule can permeate the SC either via transappendageal pathways (through hair follicles or sweat ducts), or via the transepidermal pathway (intercellular or intracellular routes). It might be expected that skin permeation of drugs in infants will be higher in comparison with that of adults. This is because of the thinner stratum corneum and epidermis in infant skin, which may result in a shorter permeation path. However, there is little information in the available literature regarding this. On the other hand, the sweat glands in newborn skin undergo functional maturation over the infancy period. How this affects the skin permeation in infants is not well understood.

The percutaneous absorption of molecules is largely dependent on the skin barrier function [74]. Some studies have reported that the skin permeation process of molecules in full-term newborns and infants is comparable to adults [74]. There is very limited information regarding systemic absorption and systemic effects resulting from the ingredients contained in baby wipes. However, one study reported that percutaneous absorption of povidone iodine used as a topical antiseptic for newborns caused impaired thyroid function in healthy full-term newborns [75]. Several cases of thyroid dysfunction in full-term newborns with spina-bifida following topical application of povidone iodine have also been reported [9,76]. In these studies, povidone iodine was applied as an antiseptic dressing once daily, but the dose applied was not disclosed. 

Some studies have reported that preterm neonates have an impaired skin barrier function and are at risk of higher exposure to chemicals [6,10,11]. These conditions potentially predispose preterm neonates to unintended systemic absorption of compounds [6,8,9,10,11,74]. Increased skin absorption of phenylephrine was observed in preterm newborns, but the absorption rate decreased as a function of postnatal age [6]. In an in vitro permeability study the skin of preterm newborns was shown to be more permeable to alcohol (up to 50 fold) and salicylates (up to 1000 fold) compared with full-term newborns [3,11]. Toxicity resulting from systemic absorption of alcohol from topical products in a preterm newborn has also been reported [10]. The skin of the baby (born at a gestational age of 27 weeks) was cleansed using methylated spirit (95% ethanol and 5% wood naphta). The post-mortem blood concentration of ethanol was 3 mg/mL [10]. A further study reported systemic absorption of chlorhexidine gluconate following the application of a 1% ethanol solution to the umbilical cord area of newborns [8]. Systemic absorption was not observed when a powder formulation of chlorhexidine gluconate was applied. Following 9 days treatment with chlorhexidine 1% in ethanol, chlorhexidine was detected in the blood. Plasma concentrations of chlorhexidine in the preterm newborns were significantly higher compared with full term newborns (*p* < 0.001). The mean plasma concentration of chlorhexidine in preterm newborns increased from 10 ng/mL at 5 days to 32 ng/mL at 9 days [8].

Although similar levels of skin absorption of compounds are reported for full term infants and adults, there are several risk factors that may contribute to an impaired skin barrier and altered percutaneous absorption in infants [1,77,78]. Among the risk factors are a higher ratio of skin surface area to body weight, limited metabolism, and the use of diapers [78,79,80].

### 4.1. Surface Area

The ratio of skin surface area to body weight in newborns is 2.3 times higher than in adults [81]. The surface area-to-body weight ratio is even higher in preterm newborns and low birthweight newborns. Skin care products such as cleansing lotions, moisturizers and sunscreens are often applied over a large body surface area. Therefore, the number and amount of topical products applied to infant skin needs to be carefully considered in order to prevent excessive percutaneous absorption of actives.

### 4.2. Pharmacokinetics and Metabolism

In newborns, the pharmacokinetic profiles of drugs (Table 3) are distinctly different compared with adults—generally characterized by longer biological half-lives (3–9 times longer) and lower clearance rates [81]. However, renal and hepatic metabolism develops rapidly during the first month after birth, and thus the pharmacokinetic and metabolic profiles of drugs become comparable to adults [81].

### 4.3. Use of Diapers

Visscher et al. (2000) studied the changes in skin physiology of newborns, particularly in the diaper area [47]. 31 full-term newborns wore diapers of similar brands and sizes. Skin hydration and pH were measured periodically (1, 4, 7, 14, 21, and 28 days) on the diaper area and non-diaper area. For the diaper area, the site of measurement was the area above the symphysis pubis (below the waistband of the diaper). The measurements were performed 25 min after the diaper was removed. The area above the waistband was chosen as the site of measurement for the non-diaper area. Skin hydration was determined by measuring capacitive reactance. To assess the water sorption-desorption, the skin was wetted with deionized water (using a gauze pad for 10 s) and blotted dry. Measurement of capacitive reactance was then performed continuously for 1 min at 10 s intervals (dynamic hydration measurement). Water sorption was assessed by comparing the capacitive reactance at the baseline (before wetting) and immediately after skin blotting. The water desorption was assessed based on the slope of the desorption curve from the dynamic hydration measurement. To measure the skin pH, a pH meter with a planar electrode was placed in contact with the skin for 3 s. The electrode was wetted with deionized water before the measurement. The authors reported that the area covered by the diaper exhibited higher surface hydration and pH values compared with the uncovered area (*p* < 0.05) [47]. This was observed 14 days after birth. The rate of water desorption both in the diaper and non-diaper area at 14 days was lower compared with the baseline (day 1). The authors suggested that this finding indicated an improved water sorption-desorption function. At the end of the neonatal period (28 days), skin hydration and water sorption-desorption functions in the diaper area were not significantly different compared with the non-diaper area. However, the reported skin pH in the diaper area remained higher compared with the non-diaper area (*p* < 0.05) [47]. 

**Figure 4:**
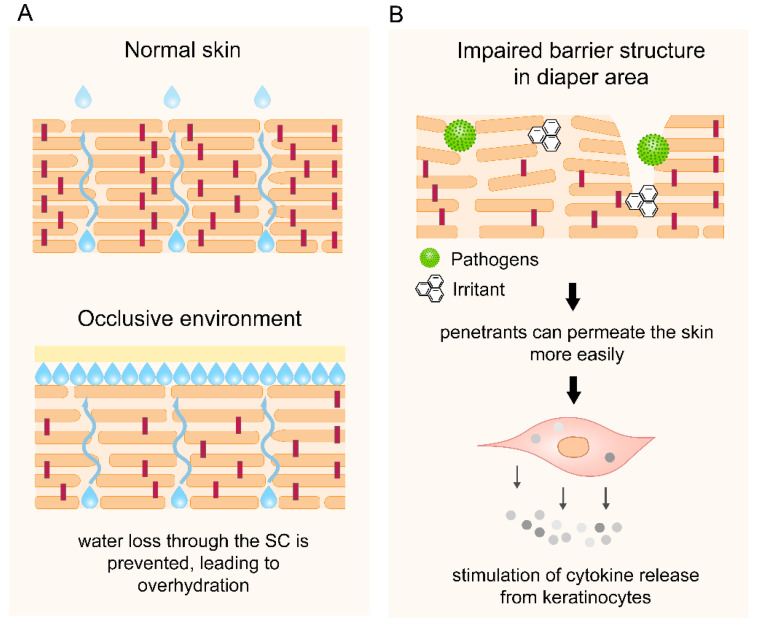
Skin permeability disruption of the diaper area. Occlusion of the SC (**A**) induced impaired skin barrier and increased skin permeability of penetrants (**B**), leading to an immunological response.

Higher skin pH for the diaper area compared with the non-diaper area has been attributed to skin over-hydration because of coverage by the diaper cloth and contact with urine and faeces [47]. The use of diapers creates an occlusive environment (Figure 4), along with trapped moisture from urine and/or faeces [84]. Newborns excrete urine every 1 to 3 h on average [85] but the frequency decreases to 7 times a day by the end of infancy [86]. Infrequent diaper changing may cause prolonged exposure to moisture, resulting in over-hydration and subsequently impairment of SC permeability (Figure 4A) [21,87]. Moreover, friction between the diaper cloth and the over-hydrated skin, particularly around the genitalia, the buttocks and skin folds, can cause further damage to the SC, or maceration [85,87]. As a result, skin permeability increases and compounds may permeate into the viable epidermis (Figure 4B), leading to irritation [3].

In addition, infant faeces contain a high concentration of digestive enzymes [88,89,90]. A study reported higher proteolytic activity of faeces (*p* < 0.01) in infants (4–12 months old) compared with children (1–8 years old) [88]. An in vivo study reported skin barrier disruption and irritation after application of faecal concentrations of proteolytic and lipolytic digestive enzymes (elastase, chymotrypsin, trypsin, and lipase) in bile salt mixtures [90]. The mixture was applied to the back of healthy adults using a Hill Top^®^ chamber [90]. Exposure to the test solutions was maintained for 21 days. TEWL was determined using an open-chamber Evaporimeter and skin pH measurements were performed according to the principles described earlier. The irritation score was determined visually. After 5 days, it was found that TEWL, skin pH, and erythema scores in groups treated with faecal enzymes significantly increased (*p* < 0.05) compared with the control group (treated with phosphate buffer pH 8) [90]. 

Several studies reported an increased pH in the skin area affected by diaper dermatitis (DD) compared with the unaffected skin area [84,91,92]. However, work by Andersen et al. (1994) suggested that the skin irritation was caused by faecal enzymes rather than by the increased skin pH [90]. The main findings in this study will be discussed in a later section. In addition, a recent review by Atherton (2016) suggested that skin maceration is the main causative factor of DD [87].

## 5. Appropriate Care of the Diaper Area

As described previously, the risk factors arising from the use of diapers potentially compromise the skin barrier function and may ultimately lead to diaper dermatitis (DD). Appropriate care of the diaper area involves (1) frequent changes of the diaper, (2) the use of effective but mild cleansers, and, if necessary, (3) application of barrier-restoring topical products [85,93]. It has been widely reported that poor management of diaper dermatitis may lead to *Candida albicans* infections, particularly in severe cases [6,94]. Topical steroids may be required in these circumstances, in combination with antifungal agents if the symptoms of infection persist [95].

## 6. Skin Cleansing of the Diaper Area

The main goal of cleansing the diaper area is to remove skin contaminants without compromising the skin barrier [96]. It has also been suggested that these formulations should provide restoration of the skin pH and the SC barrier [97]. As discussed earlier, it should be noted that findings regarding skin pH should be considered with caution. For skin cleansing purposes, various agents have been used: water, soap, liquid cleansers, and lotion-impregnated cleansing materials (baby wipes).

Cotton wool and water are commonly used by the caregiver to clean the diaper area. Water rinsing in combination with baby wipes is also widely used [97]. A recent study showed that water alone is not sufficient to remove faecal material from the diaper area [98]. To address this, baby cleansing wipes were developed and have rapidly replaced traditional cleansing practices [19]. In 2009, Proctor and Gamble noted that more than 90% of caregivers in the United States, United Kingdom, and Russia used baby wipes [99]. In addition, it has been reported that 83% of caregivers in France preferred to use baby wipes over other methods of cleaning the diaper area [99]. According to a recent market research report the use of baby wipes is increasing with a market growth of 81.73 million USD over a forecast period from 2019 to 2024 [100].

### 6.1. Baby Wipes

Baby wipes comprise of a piece of non-woven cloth that has been impregnated with a water-based cleansing emulsion intended for skin cleansing. Clinical evidence has shown the safety and effectiveness of baby wipes for infants, including preterm infants during the first month after birth [101,102]. A number of studies have provided insights into the effects of baby wipes on infant skin physiology [98,103,104,105,106,107]. All studies compared the values of skin barrier parameters in the diaper region of newborns a few weeks after birth (1–4 week) compared with baseline conditions (24–48 h after birth). Biophysical measurements were performed in environmentally controlled conditions. Use of moisturizers on the diaper region was avoided, the frequency of bathing was limited to twice a week, and the measurements were taken 30 min after the diaper was removed. As summarized in Table 4, the most noticeable findings reported are lower pH, TEWL values, and erythema scores in the baby wipes group compared with the water-and-cloth group.

The clinical studies reviewed used different methods and instruments to measure physiological conditions of the skin (erythema, hydration, skin pH, and TEWL). Validated methods and controlled measurement conditions are critical for proper interpretation and comparison of the findings in these studies. However, information regarding these aspects is lacking in some of the studies included here. Notably, a specific time period is required before measuring TEWL and skin pH [46]. This is important to anticipate dynamic changes on the skin, such as water evaporation, after removal of occlusive diapers or after application of baby wipes [106]. Most of the studies did not include an evaluation of whether the time taken for acclimatization of the skin to the observation environment was sufficient. 

In addition, some of the reports reviewed here did not acknowledge the limitations of the methods employed. For example, achieving accurate readings from TEWL measurements on infant skin using an open-chamber probe can be very challenging. This is because infants may experience distress and make unanticipated movements during the measurement [13]. At the same time, open-chamber instruments should be held horizontally to avoid interference from air convection [46,71,72]. Closed-chamber TEWL measurement instruments may minimize this risk, particularly if the probe is equipped with a condenser [72,73]. Therefore, more studies with comparable methodological approaches are warranted.

We also identified a potential risk of bias in the studies listed in Table 4. Some of the data summarized here are from infants spanning a broad age range (6–24 months). One of the studies did not disclose the age of the subjects. Considering that children have different levels of skin maturity over the infancy period, critical assessment of demographic characteristics is necessary to address the potential risk of biased or flawed interpretation. 

### 6.2. Formulation of Baby Wipes

Appropriately formulated baby wipes are essential to ensure effective cleansing at each diaper change without causing adverse effects. As a general requirement, the ingredients included in baby wipe formulations should be clinically proven to be safe for use on infant skin, with special consideration of potential irritation [108]. In 2006 the European Medicines Agency (EMA) published an article related to formulations for the paediatric population [79]. The importance of using excipients that have demonstrated safety and effectiveness for this group was highlighted. In addition, the risk of systemic absorption arising from the occlusive diaper environment in the infant population has been recognized by the EMA [79]. Despite no universal list of approved ingredients for specific use on newborns or infant skin, some ingredients (particularly surfactants, preservatives, and fragrances) are subject to regulation [79,102,108,109]. 

Most baby wipe formulations contain a large percentage of water, preservatives, surfactants, and emollients [102]. The rationale for each ingredient is listed in Table 5. It has been proposed that the formulation of baby wipes should aim to provide a pH buffering action to maintain healthy skin pH [102,110,111]. However, it remains to be seen whether such a ‘pH buffering action’ can be achieved and this will be discussed further in a later section. In 2016, a set of requirements regarding the selection of baby wipe ingredients was proposed by a panel of experts on infant skin care, based on current knowledge [108]. Some of the older preparations contain alcohols, but the majority of currently available baby wipe products are alcohol-free [98]. The use of alcohols such as isopropanol in baby wipes is strongly discouraged because of the irritating and drying properties of these solvents [108]. 

According to the latest directive from the EMA (Annex to the European Commission guideline on ‘Excipients in the labelling and package leaflet of medicinal products for human use’), the use of certain excipients with known potential of enhancing skin permeation, such as cyclodextrins, is prohibited for infants and children aged less than 2 years [109]. Some commercial baby wipe formulations contain fragrances with the aim of increasing consumer acceptance [102,108]. However, these excipients should be limited to regulator-approved ingredients (least likely to cause adverse events such as irritation and sensitization) [79,108]. An emerging trend in the formulation of baby wipes is designated products for infants with sensitive skin—these formulations do not contain fragrances or other additives reported to cause irritation [103]. Furthermore, clinical studies support the use of such formulations for infants with skin conditions such as atopic dermatitis [103,104]. 

#### 6.2.1. Water

The cleansing lotion in baby wipes contains more than 90% water [112]. Regulatory bodies have established guidelines for the quality of water for pharmaceutical use [113,114]. For example, the EMA and the United States Food and Drug Administration (FDA) have specified that the water to be used as an excipient for non-sterile dosage forms should conform to the specifications for purified water [113,114,115,116]. 

#### 6.2.2. Preservatives

Baby wipes contain a large percentage of water, and such an environment supports the growth of microorganism. It is worth noting that infants with skin conditions are more at risk from microorganisms, both pathogenic and non-pathogenic [102]. Preservatives can effectively prevent microbial growth during the shelf life of the product. 

The use of preservatives in baby wipe formulations should conform to the relevant official regulations [79,109]. According to the directive set out by the EMA in 2006, the use of benzyl alcohol, benzoic acid, and benzoate for newborns should be avoided because the metabolic pathway of these compounds is not fully developed [79]. Benzyl alcohol has been known to cause local irritation in adults [109]. Furthermore, increased risk of jaundice in newborns following skin exposure to benzoic acid and benzoates has been reported [109]. Recently, Hamann et al. (2019) reviewed the most frequently used preservatives in baby wipes in North America [117]. The top five ingredients are listed in Table 6.

Attention has been given to skin sensitization in the diaper area by *formaldehyde-releasing* preservatives [102]. Among the most common ingredients are 1,2-dimethylol-5,6-dimethylhydantoine (DMDM hydantoin), diazolidinylurea, imidazolidinylurea, methylisothiazolinone, and quaternium-15 [118,119]. Methylisothiazolinone (MI) was previously used in several commercial baby wipe products from 2005 until 2014 [120]. However, a high rate of MI sensitization was reported by the North American Contact Dermatitis Group (NACDG) in 2014 [120]. Similarly, Chang and Nakrani (2014) reported allergic contact dermatitis in children following contact with MI-containing wipes in the perianal area [121]. Interestingly, a year before these reported cases The American Contact Dermatitis Society had raised the awareness of the allergenic potential of MI by naming it as the Allergen of the Year in 2013 [119,122,123]. In response to these concerns, the range of preservatives used in disposable wipes and other leave-on products began to change [102,119]. Most baby wipe brands do not include MI, although another formaldehyde-releaser, DMDM hydantoin, is still found in some brands [117,119].

#### 6.2.3. Surfactants

In baby wipe formulations, surfactants provide a cleaning action. Surfactants are composed of hydrophilic moieties (often referred to as head groups) and hydrophobic chains. Surfactants may be classified based on the charge of the head groups: anionic (negatively charged), cationic (positively charged), non-ionic (no charge), and amphoteric (zero net charge). Non-ionic surfactants are recognized as having the mildest actions on the skin, having the lowest potential for irritation compared with other types of surfactants [102,124,125]. However, this is only confirmed for adult skin and information is lacking for infant skin. 

Surfactants may reside on the surface of the skin and interact with SC components [21]. Depending on the frequency and period of contact, the interaction between the surfactant and the SC may eventually result in skin irritation. Therefore, the amount of surfactant remaining following skin cleansing must be considered. Special attention should be given to residual amounts of surfactant in skin creases and folds. In order to minimize any irritant effects, wipe formulations for infant skin should only use surfactants with low irritation potential. As shown in Table 7, the most commonly used surfactants in baby wipe formulations belong to the non-ionic class. In contrast, rinse-off liquid cleansers typically contain anionic surfactants because of their high foaming and lathering capacity [125]. 

As noted earlier, baby wipes consist of fabric impregnated with a cleansing lotion. The cleansing lotion typically contains not more than 0.3% *w*/*w* of surfactant [102,126]. In contrast, rinse-off cleansing products for infants may contain up to 5% surfactant, considering that dilution occurs upon use [126]. For instance, bathing of infants using rinse-off skin cleansers includes washing and rinsing steps [129]. During the washing step, the cleanser is lathered and deposited on the wet skin surface. In the next step, the cleanser is removed by rinsing with water. Therefore, the concentration of surfactant in the cleansers becomes diluted sequentially. 

#### 6.2.4. Emollients

With reference to baby wipes emollients are ingredients that can help to minimize SC barrier disruption by (1) avoiding frictional damage of the skin during wiping [101], (2) ameliorating the effects of interaction of surfactants and SC components (particularly lipids and proteins), and (3) replenishing the SC lipids that are lost during cleansing [125]. The use of baby wipes impregnated with emollients was reported to result in lower erythema scores and TEWL in infants (*p* < 0.05) compared with the use of cloth and water [106]. 131 infants were subjected to one of the following treatments for 7 days: (1) cleansing of the diaper area by water and cloth or (2) emollient-containing baby wipes. The erythema score was determined visually and TEWL was determined using a closed chamber device 20 min after diaper removal. At the end of the study, erythema scores and TEWL values in the perianal area were significantly lower (*p* < 0.05) in the group treated with emollient-containing baby wipes [106]. Among the most commonly used emollient ingredients found in baby wipes are mineral oil, sorbitan sesquioleate [21], glycerine, glyceryl oleate [98], volatile silicone, and behenyl alcohol [130]. In addition to emollients, various agents that are thought to promote skin health such as vitamin E derivatives are often included in baby wipe formulations [102]. However, evidence is lacking to support the benefits of such agents for infant skin. 

#### 6.2.5. pH Buffering Compounds

The majority of commercial baby wipe formulations contain organic acids and conjugate bases (such as citric acid and sodium citrate). Several authors have endorsed the use of these ingredients in baby wipes [101,102,108]. This is based on the perceived potential impact of the occlusive environment and exposure to urine and faeces on the skin pH of the diaper area [101,103,105]. Adam et al. (2009) investigated whether baby wipes containing these components can modulate the skin pH [105]. The skin pH in the diaper area of infants was measured immediately after defecation and every 30 s after cleansing with the test baby wipes. Water-and-cloth was used for the control group. Immediately after cleansing, a temporary decrease in skin pH relative to the baseline was observed for the wipe-treated group. However, this was followed by a gradual increase in skin pH. Skin pH observed in the water-and-cloth-treated group continuously decreased over 6 min but the value was significantly higher than in the wipe-treated group (*p* < 0.05). The authors claimed that inclusion of buffer components in baby wipes is expected to counterbalance the alkalinity of the excretory products. However, the possibility of consecutive dilution at the measurement site with the wetted pH-meter probe during the measurement was not addressed. 

Regular use of “pH-balanced” baby wipes has been reported to maintain better the skin pH in infants within the normal range compared with water and cloth alone [106]. In this study, infants wore diapers of similar brands and used the test wipes for 14 days. Skin pH was measured 15–20 min after diaper change/cleansing. Lower skin pH (*p* < 0.05) was reported in the group treated with baby wipes buffered at pH 4.0 compared with the control group. Again, whether these skin pH values should really be accepted and interpreted as appropriate measurements of the skin is a topic that merits further discussion. 

### 6.3. Post-Cleansing Barrier-Repair Creams

Application of topical creams to the diaper area following cleansing is a common practice for the infant skin care regime [87]. The aim of this is to minimize friction between the skin and diaper, as well as limit skin exposure to urine and faeces [131,132]. Many formulations contain zinc oxide, a skin astringent that exhibits mild anti-inflammatory effects [19]. However, there are limited data supporting the effectiveness of zinc oxide ointment in preventing diaper dermatitis [133,134]. 

Emerging evidence supports the use of dexpanthenol at 5% to alleviate the symptoms of diaper dermatitis [135,136,137]. A number of studies showed that dexpanthenol improved the skin barrier function, based on a reduction in TEWL [135,136]. As an analog of pantothenic acid, dexpanthenol readily permeates the skin and undergoes biotransformation to pantothenic acid—a component of coenzyme A [138]. The synthesis of skin lipids is catalysed by coenzyme A [139,140]. Therefore, pantothenic acid was suggested to play an important role in this process [135]. In addition, it has been suggested that the hygroscopic nature of dexpanthenol contributes to improved skin hydration and TEWL [135]. However, studies confirming this hypothesis are lacking.

## 7. Safety Concerns of Repeated Exposure to Baby Wipes

Over the last decades, baby wipe formulations have been improved by removing ingredients with high irritation and allergenicity potential [102,117]. The range of preservatives used has been refined, and allergens such as formaldehyde releasers have been removed [102,103,119]. The use of fragrances is usually avoided or minimized [87]. Notably, the safety and effectiveness of baby wipes for infants, both full term and preterm infants, have been confirmed (Table 4). However, safety evaluation of baby wipes has largely been conducted on healthy infants. 

Furthermore, considering that the practice of wiping the diaper area is carried out regularly, the repeated exposure of the skin to ingredients contained in baby wipes is a concern. These matters are likely to be of critical importance for infants with underlying skin conditions such as DD and atopic dermatitis (AD). However, literature to support this is limited. Whether repeated use of baby wipes can potentially result in a higher accumulative concentration of baby wipe ingredients in the systemic circulation has not been explored to date. In addition, how baby wipe formulations can be formulated to provide long-term benefits for infants with DD and AD seems an obvious area to investigate but we are unaware of any ongoing work in this area. 

### 7.1. Diaper Dermatitis (DD)

DD refers to an inflammation of the skin area that is covered by a diaper [141,142]. Clinical characteristics of DD vary, including erythema and skin lesions [143]. DD during infancy is very common, with the highest prevalence at 9–12 months after birth [102,110,144]. More than 50% of the infant population reportedly have at least one episode of DD [145]. Skin exposure to faecal enzymes, that can be exacerbated by the occlusive environment in the diaper area, has been identified as the leading cause of DD [87,102,143,144,146]. Ineffective care of the diaper area, such as infrequent diaper change or insufficient cleansing, may exacerbate this condition [102].

Stamatas et al. (2011) investigated skin barrier status in infants with DD [146]. It was found that the skin area affected with mild or moderate DD showed a lower barrier integrity in comparison with unaffected skin of the diaper area (*p* < 0.05), based on TEWL measurements (closed-chamber) [146]. This finding suggests that there is a risk of increased skin permeability in infants with DD. One study reported systemic absorption of miconazole nitrate from the diaper area of infants with moderate to severe DD [147]. Infants were treated with either 0.25% miconazole nitrate ointment or 2% miconazole nitrate cream (5 times a day for 7 days, on average). Blood levels of miconazole nitrate up to 3.8 ng/mL were observed in the 0.25% miconazole nitrate ointment group and from 5–7 ng/mL for treatment with the 2% miconazole nitrate cream. However, the authors did not confirm the dose applied and the use of a control group was also not clarified.

As noted, exposure to ingredients in baby wipes on infants with DD should be of high concern. This is because of the summation of contributing factors: over hydration/maceration, frictional damage, and skin contamination around the diaper area. Critically, there is no actual experimental evidence to support this. The EU Scientific Committee on Consumer Safety (SCCS) has recognized that the safety of leave-on products for infants and children needs to be addressed [78]. In 2021 the SCCS announced the latest version of Guidance for the Testing of Cosmetic Ingredients for their Safety Evaluation [78]. One of the directions concerns an additional requirement for infant skin care products that are intended to be used on the diaper area. For the development and safety evaluation of such products, the potential impact of skin irritation (i.e., DD) on skin absorption of the ingredients should be taken into consideration [78]. Felter et al. (2017) proposed an approach for this assessment of potential absorption of compounds [148]. For ingredients with dermal absorption of 1–10%, a four-fold increase in dermal absorption in infant skin affected with DD should be assumed. For ingredients with dermal absorption of 10–50%, a two-fold increase in dermal absorption in affected skin should be assumed [148]. For ingredients with dermal absorption of more than 50%, modification of exposure assessment is not required. This framework was developed by taking into consideration the following parameters: the duration, severity, and extent of DD in infants. In addition, the skin affected with DD usually still retains some barrier properties. For other ingredients with unknown permeation data, an assumption of 100% dermal absorption has been acknowledged as very conservative [78,148]. 

### 7.2. Atopic Dermatitis (AD)

AD is a chronic condition and is characterized by dry and cracked skin. AD affects approximately 20% of children [141]. A review in 2012 reported an increasing prevalence of AD over the last few decades, particularly in developing countries [149]. Typically, AD presents with chronic pruritus and lichenification [150]. Hanifin and Rajka (1980) established the criteria for diagnosis of AD; these criteria have been widely used to diagnose AD in adults as well as infants and children [150]. The major features of AD are (1) pruritus, (2) lichenification either on flexural, facial or extensor regions of the body, (3) chronic relapse of dermatitis, and (4) family history of atopic disease(s) such as AD itself, asthma, and allergic rhinitis. In addition to these major features, symptoms of AD also include 23 minor features. Among these are the following: xerosis, ichtyosis, elevated serum IgE, and early age of onset. It has been reported that over 90% of AD patients have early age of onset (≤5 years) [150]. Individuals having at least three major features and three minor features are considered to have AD [150].

Increased TEWL has been reported in AD patients [151], which might be linked to increased skin permeability [152]. There is very limited information about skin barrier function in infants with AD. Two studies reported skin permeation of hydrocortisone in infants with AD [153,154]. In these studies, the severity of AD demonstrated an impact on dermal absorption of hydrocortisone following application of 1% hydrocortisone cream. The serum level of hydrocortisone was of the order: severe > moderate > mild AD [153,154]. These findings suggest that infants with AD might be at risk of unintended systemic absorption of ingredients contained in baby wipes. 

Safety evaluation of baby wipes has largely been conducted on healthy infants. On the other hand, infants with AD may have specific needs in terms of skin cleansing products. The Royal College of Paediatrics and Child Health (RCPCH) has recommended that emollient-containing cleansers should be used on infants and children with AD [155]. Clinical safety studies for baby wipes have been conducted on infants diagnosed with AD [103,105], as detailed in Table 4. In these studies, infants were subjected to use of the test wipes for cleansing the diaper area for 4 weeks. The results from both studies showed that with an average use of 9–12 wipes per day, the erythema score decreased within 4-weeks of using the test wipe in comparison with the baseline values (day 0, *p* > 0.05) [103,105]. 

## 8. Challenges and Opportunities

Studies on infant skin are challenging because of the high inter-subject variability and different maturation level of the skin over the infancy period. Furthermore, there are ethical considerations regarding in vivo studies on infants. Several non-invasive biophysical methods to assess skin functions in infants have been developed. Among these methods are TEWL, skin hydration, and pH [2,13,106]. However, non-invasive methods for assessing infant skin permeation of topically applied ingredients have not been extensively explored. 

Future opportunities for this field of research should include the application of Confocal Raman Spectroscopy (CRS) for in vivo assessment of percutaneous absorption of chemicals in infants. This method has demonstrated the ability to provide information on spatial distribution of various compounds in adult skin [156,157,158,159]. The use of CRS for studies on infants has been reported by Nikolovski et al. (2008) [13]. The authors used CRS to measure skin hydration and NMF concentrations in infants. Recently, Stamatas et al. (2021) proposed a computational model for estimation of permeation of actives in infant skin [160]. Briefly, skin permeation of a marker compound (caffeine) in infants and adults was determined using CRS. The middle of the ventral side of volar forearm was chosen for the measurements. The results obtained using the computational model compared very well with the experimental data [160]. This technique clearly has potential for evaluation of chemical permeation following repeated use of baby wipes and other infant skin care products.

## 9. Conclusions

The present review has considered the skin barrier function in young children with particular attention focused on how care of the diaper area might impact on skin integrity. Clinical studies have confirmed the safety and effectiveness of baby wipes in comparison to the use of water and cloth. Most studies to date have reported parameters such as pH, TEWL, skin hydration and erythema score. We believe the value of studies that measure pH is limited; the results reported do not reflect the actual quantitative meaning and understanding of this term. However, information regarding systemic absorption of the ingredients contained in baby wipes through the skin is lacking. It is clear that permeation of ingredients commonly found in baby wipes is worth investigating. Such information is particularly useful, considering that baby wipes are used regularly in infants. In addition, there are specific infant populations with underlying skin conditions. Considering the practice of regular skin cleansing by using baby wipes, skin barrier function and permeability in this group require special attention. However, conventional permeation studies on infant skin are difficult to conduct because of ethical and safety issues. A promising future direction in this research field is the use of emerging non-invasive spectroscopic methods. 

## Figures and Tables

**Table 1:** Nomenclature for newborn babies.

Category	Definition
Neonatal/newborn	the first 4 week after birth
Infancy	the whole first year after birth
Full-term infants	infants born between 37th–42nd week of gestation age
Preterm infants	infants born before the 37th week of gestation age
Low birth-weight infants	infants born with a birth weight of lower than 2.5 kg

**Table 2:** Skin physiology of healthy infants in comparison to adults.

Parameters	Properties
**Structure**	
Epidermis—cell size	Smaller corneocytes and keratinocytes [2]
Epidermis—surface	Higher density of skin microrelief network [2]
Epidermis—thickness	SC: 30% thinner [36]; Epidermis: 20% thinner [2]
Dermis—organization	More homogenous dermal papilla [2]; Extensive but disorganized vascular network [37]; Lower density of collagen fiber bundles [37]
**Composition**	
NMF	Lower [13]
Melanin	Lower [38]
Water	Lower at birth, gradually increasing throughout the first year [2]
**Function**	
Cell turnover	Higher [2]
Hydration and water-holding capacity	Lower hydration at birth, peaks between 3–12 months [13]; lower water holding capacity [2]
Immunological barrier	Epidermal LC are not fully mature [38]
pH	Higher [2]
Photoprotection	Melanocytes are not fully mature [38]
Sebaceous activity	Higher at birth; decreases drastically within the first few days [27]
TEWL	Higher at birth, gradually decreases throughout the first few years [38]

**Table 3:** Pharmacokinetic parameters in healthy newborns compared with adults [81,82,83].

Parameters	Properties
Blood-brain barrier	Less developed
Conjugation reactions	Lower rate
Cytochrome P450 biotransformation	Lower rate
Glomerular filtration	Lower rate
Liver mass	Higher
Plasma protein binding	Lower
Water content per body weight	Higher

**Table 4:** Summary of clinical studies comparing the use of baby wipes versus water and wool/cloth.

Parameter	Findings	Subject	Reference
pH	pH was comparable ** in both groups	Newborns (*n* = 280)	[98]
	Higher * skin pH in water-and-cloth group	Infants (*n* = 15)	[105]
	pH was comparable ** in both groups	Newborns (*n* = 44)	[107]
Hydration	Skin hydration was comparable ** in both groups	Newborns (*n* = 280)	[98]
	Skin hydration was comparable ** in both groups	Newborns (*n* = 44)	[107]
TEWL	TEWL was comparable ** in both groups	Newborns (*n* = 280)	[98]
	Lower * TEWL in baby wipes group	Newborns (*n* = 44)	[107]
Erythema	Erythema score around genitals, perianal area, and buttock was comparable* in both groups; lower* erythema score around the skin folds in baby wipes group	Infants (*n* = 102)	[103]
	lower * erythema score in baby wipes group	Preterm infants (*n* = 130)	[106]
	lower * erythema score around perianal area in baby wipes group	Infants (*n* = 82)	[104]
	IL-1α expression was comparable ** in both groups	Newborns (*n* = 44)	[107]

* statistically significant, ** statistically insignificant.

**Table 5:** Suggested requirements for components of baby wipe formulations.

Ingredient	Rationale	Recommendation
Water	As the vehicle for the cleansing ingredients [102]	Highly purified [102]
Preservatives	To prevent microbial growth in the products [102]	Should not alter the normal cutaneous microbiome; use only regulator-approved ingredients [108]
Surfactants	Water alone is ineffective in removing water-insoluble skin soil; for optimal cleaning [102]	Should effectively remove faeces and urine; should not include harsh surfactants, particularly sodium lauryl sulphate [108]
Emollients	To minimize friction and to replenish the SC lipids [102]	Should exert positive effect on skin barrier function [108]
pH buffering compounds	To maintain a healthy skin pH [102]	Should maintain the skin pH at ±5.5 [108]

**Table 6:** Preservative ingredients commonly found in baby wipes in 2019, adapted from [117].

Ingredient	Frequency (%) ^1^
Sodium citrate/citric acid	69
Sodium benzoate	62
Phenoxyethanol	48
Iodopropyl butylcarbamate	23.5
Ethylhexylglycerin	6.5

^1^ average from two retailers.

**Table 7:** Common surfactants used in baby wipe formulations.

Surfactant	Type	Typical Concentration (% *w*/*w*)
Coco-betaine (cocoamidopropyl betaine)	Amphoteric	<0.5% [126]
Coco-glucoside, or decyl glucoside, or lauryl glucoside	Non-ionic	<0.5% [126]
Glyceryl stearate	Non-ionic	1.0–2.0% [127]
Glyceryl stearate citrate	Anionic	0.5–2.0% [127]
PEG-40 hydrogenated castor oil	Non-ionic	<0.8% [128]
Polysorbate 20	Non-ionic	<0.5% [126]
Sodium cocoamphoacetate or disodium cocoamphodiacetate	Amphoteric	<0.5% [126]

## Data Availability

Not applicable.

## References

[1] West D.P., Worobec S., Solomon L.M. (1981). Pharmacology and toxicology of infant skin. J. Investig. Dermatol..

[2] Stamatas G.N., Nikolovski J., Mack M.C., Kollias N. (2011). Infant skin physiology and development during the first years of life: A review of recent findings based on in vivo studies. Int. J. Cosmet. Sci..

[3] Barker N., Hadgraft J., Rutter N. (1987). Skin permeability in the newborn. J. Investig. Dermatol..

[4] Chiou Y.B., Blume-Peytavi U. (2004). Stratum corneum maturation. Skin Pharmacol. Physiol..

[5] Hammarlund K., Sedin G. (1979). Transepidermal water loss in newborn infants: III. Relation to gestational age. Acta Paediatr..

[6] Harpin V., Rutter N. (1983). Barrier properties of the newborn infant’s skin. J. Pediatr..

[7] West D.P., Halket J.M., Harvey D.R., Hadgraft J., Solomon L.M., Harper J.I. (1987). Percutaneous absorption in preterm infants. Fetal Neonatal Investig. Rep..

[8] Aggett P.G., Cooper L.V., Ellis S.H., McAinsh J. (1981). Percutaneous absorption of chlorhexidine in neonatal cord care. Arch. Dis. Child..

[9] Aitken J., Williams F.L. (2014). A systematic review of thyroid dysfunction in preterm neonates exposed to topical iodine. Arch. Dis. Child..

[10] Harpin V., Rutter N. (1982). Percutaneous alcohol absorption and skin necrosis in a preterm infant. Arch. Dis. Child..

[11] McCormack J.J., Boisits E.K., Fisher L.B., Maibach H.I., Boisits E.K. (1982). An in vitro comparison of the permeability of adult versus neonatal skin. Neonatal Skin: Structure and Function.

[12] Fairley J.A., Rasmussen J.E. (1983). Comparison of stratum corneum thickness in children and adults. J. Am. Acad. Dermatol..

[13] Nikolovski J., Stamatas G.N., Kollias N., Wiegand B.C. (2008). Barrier function and water-holding and transport properties of infant stratum corneum are different from adult and continue to develop through the first year of life. J. Investig. Dermatol..

[14] Giusti F., Martella A., Bertoni L., Seidenari S. (2001). Skin barrier, hydration, and pH of the skin of infants under 2 years of age. Pediatr. Dermatol..

[15] Schade H., Marchionini A. (1928). Der säuremantel der haut (nach gaskettenmessungen). Klin. Wochenschr..

[16] Warner R.K., Bush R.D., Ruebusch N.A. (1995). Corneocytes Undergo Systematic Changes in Element Concentrations Across the Human Inner Stratum Corneum. J. Investig. Dermatol..

[17] Schmid-Wendtner M.H., Korting H.C. (2006). The pH of the Skin Surface and Its Impact on the Barrier Function. Skin Pharmacol. Physiol..

[18] Rothman S. (1954). pH of sweat and skin surface. Physiology and Biochemistry of the Skin.

[19] Lemper M., De Paepe K., Rogiers V., Adam R., Barel A.O., Paye M., Maibach H.I. (2009). Baby Care Products. Handbook of Cosmetic Science and Technology.

[20] McGrath J., Eady R., Pope F. (2004). Anatomy and organization of human skin. Rook’s Textbook of Dermatology.

[21] Visscher M.O. (2009). Update on the use of topical agents in neonates. Newborn Infant. Nurs. Rev..

[22] Ananthapadmanabhan K., Mukherjee S., Chandar P. (2013). Stratum corneum fatty acids: Their critical role in preserving barrier integrity during cleansing. Int. J. Cosmet. Sci..

[23] Elias P.M. (1983). Epidermal lipids, barrier function, and desquamation. J. Investig. Dermatol..

[24] Wertz P.W., van den Bergh B. (1998). The physical, chemical and functional properties of lipids in the skin and other biological barriers. Chem. Phys. Lipids.

[25] Feingold K.R. (2007). Thematic review series: Skin lipids. The role of epidermal lipids in cutaneous permeability barrier homeostasis. J. Lipid Res..

[26] Candi E., Schmidt R., Melino G. (2005). The cornified envelope: A model of cell death in the skin. Nat. Rev. Mol..

[27] Oranges T., Dini V., Romanelli M. (2015). Skin physiology of the neonate and infant: Clinical implications. Adv. Wound Care.

[28] Rawlings A.V., Matts P.J. (2005). Stratum corneum moisturization at the molecular level: An update in relation to the dry skin cycle. J. Investig. Dermatol..

[29] Burgeson R.E., Christiano A.M. (1997). The dermal-epidermal junction. Curr. Opin. Cell Biol..

[30] Fluhr J.W., Darlenski R., Taieb A., Hachem J.P., Baudouin C., Msika P., De Belilovsky C., Berardesca E. (2010). Functional skin adaptation in infancy—Almost complete but not fully competent. Exp. Dermatol..

[31] Hoeger P.H., Schreiner V., Klaassen I.A., Enzmann C.C., Friedrichs K., Bleck O. (2002). Epidermal barrier lipids in human vernix caseosa: Corresponding ceramide pattern in vernix and fetal skin. Br. J. Dermatol..

[32] Haubrich K.A. (2003). Role of vernix caseosa in the neonate: Potential application in the adult population. Am. Assoc. Crit.-Care Nurses Clin. Issues.

[33] Bautista M.I.B., Wickett R.R., Visscher M.O., Pickens W.L., Hoath S.B. (2000). Characterization of Vernix Caseosa as a Natural Biofilm: Comparison to Standard Oil-Based Ointments. Pediatr. Dermatol..

[34] Walker L., Downe S., Gomez L. (2005). Skin care in the well term newborn: Two systematic reviews. Birth.

[35] World Health Organization (2003). Pregnancy, Childbirth, Postpartum, and Newborn Care: A Guide for Essential Practice.

[36] Stamatas G.N., Nikolovski J., Luedtke M.A., Kollias N., Wiegand B.C. (2010). Infant skin microstructure assessed in vivo differs from adult skin in organization and at the cellular level. Pediatr. Dermatol..

[37] Vitellaro-Zuccarello L., Cappelletti S., Dal Pozzo Rossi V., Sari-Gorla M. (1994). Stereological analysis of collagen and elastic fibers in the normal human dermis: Variability with age, sex, and body region. Anat. Rec..

[38] Silverman R.A., Schachner L.A., Hansen R.C. (2014). Pediatric Dermatology.

[39] Lund C., Baran R., Maibach H. (1994). Newborn skin care. Cosmetic Dermatology.

[40] Hardman M.J., Byrne C., Hoath S.B., Maibach H.I. (2003). Skin structural development. Neonatal Skin: Structure and Function.

[41] Hoeger P.H., Enzmann C.C. (2002). Skin physiology of the neonate and young infant: A prospective study of functional skin parameters during early infancy. Pediatr. Dermatol..

[42] Mohammed D., Matts P.J., Hadgraft J., Lane M.E. (2012). Variation of Stratum Corneum Biophysical and Molecular Properties with Anatomic Site. AAPS J..

[43] Roberts D., Marks R. (1980). The Determination of Regional and Age Variations in the Rate of Desquamation: A Comparison of Four Techniques. J. Investig. Dermatol..

[44] Machado M., Hadgraft J., Lane M.E. (2010). Assessment of the variation of skin barrier function with anatomic site, age, gender and ethnicity. Int. J. Cosmet. Sci..

[45] Machado M., Salgado T.M., Hadgraft J., Lane M.E. (2010). The relationship between transepidermal water loss and skin permeability. Int. J. Pharm..

[46] Rogiers V. (2001). EEMCO Guidance for the Assessment of Transepidermal Water Loss in Cosmetic Sciences. Skin Pharmacol. Appl. Skin Physiol..

[47] Visscher M.O., Chatterjee R., Munson K.A., Pickens W.L., Hoath S.B. (2000). Changes in diapered and nondiapered infant skin over the first month of life. Pediatr. Dermatol..

[48] Saijo S., Tagami H. (1991). Dry skin of newborn infants: Functional analysis of the stratum corneum. Pediatr. Dermatol..

[49] Capone K., Dowd S., Stamatas G., Nikolovski J. (2010). Survey of bacterial diversity on infant skin over the first year of life. Journal of Investigative Dermatology.

[50] Stamatas G.N., Tierney N.K. (2012). Update on infant skin with special focus on dryness and the impact of moisturizers. Treatment of Dry Skin Syndrome.

[51] Wanke I., Steffen H., Christ C., Krismer B., Götz F., Peschel A., Schaller M., Schittek B. (2011). Skin commensals amplify the innate immune response to pathogens by activation of distinct signaling pathways. J. Investig. Dermatol..

[52] Parra J.L., Paye M. (2003). EEMCO Guidance for the in vivo Assessment of Skin Surface pH. Skin Pharmacol. Appl. Skin Physiol..

[53] Behrendt H., Green M. (1958). Skin pH Pattern in the Newborn Infant. AMA J. Dis. Child..

[54] Yosipovitch G., Maayan-Metzger A., Merlob P., Sirota L. (2000). Skin barrier properties in different body areas in neonates. Pediatrics.

[55] Adam R. (2008). Skin care of the diaper area. Pediatr. Dermatol..

[56] Ludriksone L., Bartels N.G., Kanti V., Blume-Peytavi U., Kottner J. (2014). Skin barrier function in infancy: A systematic review. Arch. Dermatol. Res..

[57] Deraison C., Bonnart C., Lopez F., Besson C., Robinson R., Jayakumar A., Wagberg F., Brattsand M., Hachem J.P., Leonardsson G. (2007). LEKTI fragments specifically inhibit KLK5, KLK7, and KLK14 and control desquamation through a pH-dependent interaction. Mol. Biol. Cell.

[58] Hachem J.P., Man M.Q., Crumrine D., Uchida Y., Brown B.E., Rogiers V., Roseeuw D., Feingold K.R., Elias P.M. (2005). Sustained serine proteases activity by prolonged increase in pH leads to degradation of lipid processing enzymes and profound alterations of barrier function and stratum corneum integrity. J. Investig. Dermatol..

[59] Matousek J.L., Campbell K.L. (2002). A comparative review of cutaneous pH. Vet. Dermatol..

[60] Mauro T., Holleran W.M., Grayson S., Gao W.N., Man M.Q., Kriehuber E., Behne M., Feingold K.R., Elias P.M. (1998). Barrier recovery is impeded at neutral pH, independent of ionic effects: Implications for extracellular lipid processing. Arch. Dermatol. Res..

[61] Menon G.K., Feingold K.R., Elias P.M. (1992). Lamellar Body Secretory Response to Barrier Disruption. J. Investig. Dermatol..

[62] Bouwstra J., Dubbelaar F., Gooris G., Ponec M. (2000). The lipid organisation in the skin barrier. Acta Derm. Venereol..

[63] Holleran W.M., Takagi Y., Imokawa G., Jackson S., Lee J.M., Elias P.M. (1992). β-Glucocerebrosidase activity in murine epidermis: Characterization and localization in relation to differentiation. J. Lipid Res..

[64] Mack M.C., Tierney N.K., Ruvolo E., Stamatas G.N., Martin K.M., Kollias N. (2010). Development of Solar UVR-Related Pigmentation Begins as Early as the First Summer of Life. J. Investig. Dermatol..

[65] Moise A.F., Harrison S.L., Gies H.P. (1999). Solar ultraviolet radiation exposure of infants and small children. Photodermatol. Photoimmunol. Photomed..

[66] Paller A.S., Hawk J.L.M., Honig P., Giam Y.C., Hoath S., Mack M.C., Stamatas G.N. (2011). New Insights About Infant and Toddler Skin: Implications for Sun Protection. Pediatrics.

[67] Zouboulis C.C., Fimmel S., Ortmann J., Turnbull J.R., Boschnakow A., Pochi P., Hoath S.B., Maibach H.I. (2003). Sebaceous glands. Neonatal Skin: Structure and Function.

[68] Henderson C.A., Taylor J., Cunliffe W.J. (2000). Sebum excretion rates in mothers and neonates. Br. J. Dermatol..

[69] Agache P., Blanc D., Barrand C., Laurent R. (1980). Sebum levels during the first year of life. Br. J. Dermatol..

[70] Ramasastry P., Downing D.T., Pochi P.E., Strauss J.S. (1970). Chemical Composition of Human Skin Surface Lipids from Birth to Puberty. J. Investig. Dermatol..

[71] Cohen J.C., Hartman D.G., Garofalo M.J., Basehoar A., Raynor B., Ashbrenner E., Akin F.J. (2009). Comparison of closed chamber and open chamber evaporimetry. Skin Res. Technol..

[72] Berardesca E., Loden M., Serup J., Masson P., Rodrigues L.M. (2018). The revised EEMCO guidance for the in vivo measurement of water in the skin. Skin Res. Technol..

[73] Imhof R.E., De Jesus M.E.P., Xiao P., Ciortea L.I., Berg E.P. (2009). Closed-chamber transepidermal water loss measurement: Microclimate, calibration and performance. Int. J. Cosmet. Sci..

[74] Delgado-Charro M.B., Guy R.H. (2014). Effective use of transdermal drug delivery in children. Adv. Drug Deliv. Rev..

[75] AvRuskin T.W., Greenfield E., Prasad V., Greig F., Juan C.S. (1994). Decreased T3 and T4 levels following topical application of povidone-iodine in premature neonates. J. Pediatr. Endocrinol. Metab..

[76] Barakat M., Carson D., Hetherton A.M., Symth P., Leslie H. (1994). Hypothyroidism secondary to topical iodine treatment in infants with spina bifida. Acta Paediatr..

[77] Hoath S.B., Maibach H.I. (2003). Neonatal Skin: Structure and Function.

[78] Bernauer U., Bodin L., Chaudhry Q., Coenraads P.J., Dusinska M., Ezendam J., Gaffet E., Galli C.L., Granum B., Panteri E. (2021). The SCCS Notes of Guidance for the testing of cosmetic ingredients and their safety evaluation, 11th revision, 30–31 March 2021, SCCS/1628/21. Regul. Toxicol. Pharmacol..

[79] European Medicines Agency. Reflection Paper: Formulations of Choice for the Paediatric Population. EMEA/CHMP/PEG/194810/2005. https://www.ema.europa.eu/en/documents/scientific-guideline/reflection-paper-formulations-choice-paediatric-population_en.pdf.

[80] West D.P., Heath C., Cameron Haley A., Mahoney A., Micali G. (2011). Principles of Paediatric Dermatological Therapy. Harper’s Textbook of Pediatric Dermatology.

[81] Renwick A. (1998). Toxicokinetics in infants and children in relation to the ADI and TDI. Food Addit. Contam..

[82] Dorne J. (2004). Impact of inter-individual differences in drug metabolism and pharmacokinetics on safety evaluation. Fundam. Clin. Pharmacol..

[83] Dorne J., Walton K., Renwick A. (2005). Human variability in xenobiotic metabolism and pathway-related uncertainty factors for chemical risk assessment: A review. Food Chem. Toxicol..

[84] Klunk C., Domingues E., Wiss K. (2014). An update on diaper dermatitis. Clin. Dermatol..

[85] Atherton D., Gennery A., Cant A. (2004). The neonate. Rook’s Textbook of Dermatology.

[86] Evans N., Rutter N. (1986). Development of the epidermis in the newborn. Neonatology.

[87] Atherton D.J. (2016). Understanding irritant napkin dermatitis. Int. J. Dermatol..

[88] Ruseler-van Embden J.G.H., Van Lieshout L.M.C., Smits S.A., Van Kessel I., Laman J.D. (2004). Potato tuber proteins efficiently inhibit human faecal proteolytic activity: Implications for treatment of peri-anal dermatitis. Eur. J. Clin. Investig..

[89] Berg R.W., Buckingham K.W., Stewart R.L. (1986). Etiologic Factors in Diaper Dermatitis: The Role of Urine. Pediatr. Dermatol..

[90] Andersen P.H., Bucher A.P., Saeed I., Lee P.C., Davis J.A., Maibach H.I. (1994). Faecal enzymes: In vivo human skin irritation. Contact Dermat..

[91] Yonezawa K., Haruna M., Shiraishi M., Matsuzaki M., Sanada H. (2014). Relationship between skin barrier function in early neonates and diaper dermatitis during the first month of life: A prospective observational study. Pediatr. Dermatol..

[92] Berg R.W., Milligan M.C., Sarbaugh F.C. (1994). Association of skin wetness and pH with diaper dermatitis. Pediatr. Dermatol..

[93] Benjamin L. (1987). Clinical correlates with diaper dermatitis. Pediatrician.

[94] Martin-Bouyer G., Toga M., Lebreton R., Stolley P., Lockhart J. (1982). Outbreak of accidental hexachlorophene poisoning in France. Lancet.

[95] Concannon P., Gisoldi E., Phillips S., Grossman R. (2001). Diaper dermatitis: A therapeutic dilemma—Results of a double-blind placebo controlled trial of miconazole nitrate 0.25%. Pediatr. Dermatol..

[96] Gelmetti C. (2001). Skin cleansing in children. J. Eur. Acad. Dermatol. Venereol..

[97] Coughlin C.C., Frieden I.J., Eichenfield L.F. (2014). Clinical approaches to skin cleansing of the diaper area: Practice and challenges. Pediatr. Dermatol..

[98] Lavender T., Furber C., Campbell M., Victor S., Roberts I., Bedwell C., Cork M.J. (2012). Effect on skin hydration of using baby wipes to clean the napkin area of newborn babies: Assessor-blinded randomised controlled equivalence trial. BMC Pediatr..

[99] Procter & Gamble (2009). Data on File.

[100] Technavio Wet Tissue and Wipe Market by Application, Distribution Channel, Technology, and Geography-Forecast & Analysis 2021–2025. https://www.technavio.com/report/wet-tissue-and-wipe-market-industry-analysis.

[101] Vongsa R., Rodriguez K., Koenig D., Cunningham C. (2019). Benefits of using an appropriately formulated wipe to clean diapered skin of preterm infants. Glob. Pediatr. Health.

[102] Rodriguez K.J., Cunningham C., Foxenberg R., Hoffman D., Vongsa R. (2020). The science behind wet wipes for infant skin: Ingredient review, safety, and efficacy. Pediatr. Dermatol..

[103] Ehretsmann C., Schaefer P., Adam R. (2001). Cutaneous tolerance of baby wipes by infants with atopic dermatitis, and comparison of the mildness of baby wipe and water in infant skin. J. Eur. Acad. Dermatol. Venereol..

[104] Odio M., Streicher-Scott J., Hansen R.C. (2001). Disposable baby wipes: Efficacy and skin mildness. Dermatol. Nurs..

[105] Adam R., Schnetz B., Mathey P., Pericoi M., de Prost Y. (2009). Clinical demonstration of skin mildness and suitability for sensitive infant skin of a new baby wipe. Pediatr. Dermatol..

[106] Visscher M., Odio M., Taylor T., White T., Sargent S., Sluder L., Smith L., Flower T., Mason B., Rider M. (2009). Skin care in the NICU patient: Effects of wipes versus cloth and water on stratum corneum integrity. Neonatology.

[107] Garcia Bartels N., Massoudy L., Scheufele R., Dietz E., Proquitté H., Wauer R., Bertin C., Serrano J., Blume-Peytavi U. (2012). Standardized diaper care regimen: A prospective, randomized pilot study on skin barrier function and epidermal IL-1α in newborns. Pediatr. Dermatol..

[108] Blume-Peytavi U., Lavender T., Jenerowicz D., Ryumina I., Stalder J.F., Torrelo A., Cork M.J. (2016). Recommendations from a European roundtable meeting on best practice healthy infant skin care. Pediatr. Dermatol..

[109] European Medicines Agency Annex to the European Commission Guideline on ‘Excipients in the Labelling and Package Leaflet of Medicinal Products for Human Use’ (SANTE-2017-11668). https://www.ema.europa.eu/en/annexeuropean-%20commission-guidelineexcipients-%20labelling-package-leafletmedicinal-%20products-human.

[110] Blume-Peytavi U., Hauser M., Lunnemann L., Stamatas G.N., Kottner J., Garcia Bartels N. (2014). Prevention of diaper dermatitis in infants—A literature review. Pediatr. Dermatol..

[111] Rippke F., Schreiner V., Schwanitz H.J. (2002). The acidic milieu of the horny layer. Am. J. Clin. Dermatol..

[112] Salama P., Gliksberg A., Cohen M., Tzafrir I., Ziklo N. (2021). Why are wet wipes so difficult to preserve? Understanding the intrinsic causes. Cosmetics.

[113] European Medicines Agency Guideline on the Quality of Water for Pharmaceutical Use. EMA/CHMP/CVMP/QWP/496873/2018. https://www.ema.europa.eu/en/documents/scientific-guideline/guideline-quality-water-pharmaceutical-use_en.pdf.

[114] US Food and Drug Administration. Water for Pharmaceutical Use. https://www.fda.gov/inspections-compliance-enforcement-and-criminal-investigations/inspection-technical-guides/water-pharmacuetical-use.

[115] United States Pharmacopeia (2019). USP 43-NF 38. Chapter <1231> Water for Pharmaceutical Purposes.

[116] The European Pharmacopoeia (2010). Monograph 0008. Purified Water.

[117] Hamann C.R., Sahni S., Zug K.A. (2019). Methylisothiazolinone: Still on Leave-on Products, but No Longer on Baby Wipes. Dermatitis.

[118] Varvaresou A., Papageorgiou S., Tsirivas E., Protopapa E., Kintziou H., Kefala V., Demetzos C. (2009). Self-preserving cosmetics. Int. J. Cosmet. Sci..

[119] Yu J., Treat J., Chaney K., Brod B. (2016). Potential allergens in disposable diaper wipes, topical diaper preparations, and disposable diapers: Under-recognized etiology of pediatric perineal dermatitis. Dermatitis.

[120] Zirwas M.J., Hamann D., Warshaw E.M., Maibach H.I., Taylor J.S., Sasseville D., DeKoven J.G., Fransway A.F., Mathias C.G.T., Zug K.A. (2017). Epidemic of isothiazolinone allergy in North America: Prevalence data from the North American contact dermatitis group, 2013–2014. Dermatitis.

[121] Chang M.W., Nakrani R. (2014). Six children with allergic contact dermatitis to methylisothiazolinone in wet wipes (baby wipes). Pediatrics.

[122] Cahill J.L., Toholka R.W., Nixon R.L. (2014). Methylisothiazolinone in baby wipes: A rising star among causes of contact dermatitis. Med. J. Aust..

[123] Castanedo-Tardana M.P., Zug K.A. (2013). Methylisothiazolinone. Dermatitis.

[124] Bárány E., Lindberg M., Lodén M. (2000). Unexpected skin barrier influence from nonionic emulsifiers. Int. J. Pharm..

[125] Ananthapadmanabhan K., Moore D.J., Subramanyan K., Misra M., Meyer F. (2004). Cleansing without compromise: The impact of cleansers on the skin barrier and the technology of mild cleansing. Dermatol. Ther..

[126] Cunningham C., Mundschau S., Seidling J., Wenzel S., Schlossman M. (2008). Baby care. The Chemistry and Manufacture of Cosmetics.

[127] Cunningham C.T., Seidling J.R., Kroll L.M., Mundschau S.A. (2016). Stable Emulsion for Prevention of Skin Irritation and Articles Using Same.

[128] Sheehan A.A. (2011). Non-Wovens with High Interfacial Pore Size and Method of Making Same.

[129] Draelos Z.D. (2018). The science behind skin care: Cleansers. J. Cosmet. Dermatol..

[130] Andersen F.A. (2011). Annual review of cosmetic ingredient safety assessments: 2007–2010. Int. J. Toxicol..

[131] Atherton D.J. (2004). A review of the pathophysiology, prevention and treatment of irritant diaper dermatitis. Curr. Med. Res. Opin..

[132] Scheinfeld N. (2005). Diaper Dermatitis: A Review and Brief Survey of Eruptions of the Diaper Area. Am. J. Clin. Dermatol..

[133] Garcia Bartels N., Lunnemann L., Stroux A., Kottner J., Serrano J., Blume-Peytavi U. (2014). Effect of diaper cream and wet wipes on skin barrier properties in infants: A prospective randomized controlled trial. Pediatr. Dermatol..

[134] Rowe J., McCall E., Kent B. (2008). Clinical effectiveness of barrier preparations in the prevention and treatment of nappy dermatitis in infants and preschool children of nappy age. Int. J. Evid.-Based Healthc..

[135] Gehring W., Gloor M. (2000). Effect of topically applied dexpanthenol on epidermal barrier function and stratum corneum hydration. Arzneimittelforschung.

[136] Wananukul S., Limpongsanuruk W., Singalavanija S., Wisuthsarewong W. (2006). Comparison of dexpanthenol and zinc oxide ointment with ointment base in the treatment of irritant diaper dermatitis from diarrhea: A multicenter study. J. Med. Assoc. Thai..

[137] Wolff H.H., Kieser M. (2007). Hamamelis in children with skin disorders and skin injuries: Results of an observational study. Eur. J. Pediatr..

[138] Abiko Y., Tomikawa M., Shimizu M. (1969). Enzymatic conversion of pantothenylalcohol to pantothenic acid. J. Vitam..

[139] Slyshenkov V.S., Rakowska M., Moiseenok A.G., Wojtczak L. (1995). Pantothenic acid and its derivatives protect Ehrlich ascites tumor cells against lipid peroxidation. Free Radic. Biol. Med..

[140] Proksch E., Nissen H.P. (2002). Dexpanthenol enhances skin barrier repair and reduces inflammation after sodium lauryl sulphate-induced irritation. J. Dermatolog. Treat..

[141] Telofski L.S., Morello A.P., Mack Correa M.C., Stamatas G.N. (2012). The Infant Skin Barrier: Can We Preserve, Protect, and Enhance the Barrier?. Dermatol. Res. Pract..

[142] Atherton D. (2001). The aetiology and management of irritant diaper dermatitis. J. Eur. Acad. Dermatol. Venereol..

[143] Coughlin C.C., Eichenfield L.F., Frieden I.J. (2014). Diaper Dermatitis: Clinical Characteristics and Differential Diagnosis. Pediatr. Dermatol..

[144] Stamatas G.N., Tierney N.K. (2014). Diaper Dermatitis: Etiology, Manifestations, Prevention, and Management. Pediatr. Dermatol..

[145] Adalat S., Wall D., Goodyear H. (2007). Diaper Dermatitis-Frequency and Contributory Factors in Hospital Attending Children. Pediatr. Dermatol..

[146] Stamatas G.N., Zerweck C., Grove G., Martin K.M. (2011). Documentation of Impaired Epidermal Barrier in Mild and Moderate Diaper Dermatitis In Vivo Using Noninvasive Methods. Pediatr. Dermatol..

[147] Eichenfield L.F., Bogen M.L. (2007). Absorption and efficacy of miconazole nitrate 0.25% ointment in infants with diaper dermatitis. J. Drugs Dermatol. JDD.

[148] Felter S.P., Carr A.N., Zhu T., Kirsch T., Niu G. (2017). Safety evaluation for ingredients used in baby care products: Consideration of diaper rash. Regul. Toxicol. Pharmacol..

[149] Deckers I.A.G., McLean S., Linssen S., Mommers M., van Schayck C.P., Sheikh A. (2012). Investigating International Time Trends in the Incidence and Prevalence of Atopic Eczema 1990-2010: A Systematic Review of Epidemiological Studies. PLoS ONE.

[150] Hanifin J.M., Rajka G. (1980). Diagnostic features of atopic dermatitis. Acta Derm. Venereol..

[151] Linde Y.W. (1992). Dry skin in atopic dermatitis. Acta Derm. Venereol. Suppl..

[152] Levin J., Maibach H. (2005). The correlation between transepidermal water loss and percutaneous absorption: An overview. J. Control. Release.

[153] Turpeinen M., Salo O., Leisti S. (1986). Effect of percutaneous absorption of hydrocortisone on adrenocortical responsiveness in infants with severe skin disease. Br. J. Dermatol..

[154] Turpeinen M. (1988). Influence of age and severity of dermatitis on the percutaneous absorption of hydrocortisone in children. Br. J. Dermatol..

[155] Cox H., Lloyd K., Williams H., Arkwright P.D., Brown T., Clark C., Campbell M., Gore C., Hardman C., Langford A. (2011). Emollients, education and quality of life: The RCPCH care pathway for children with eczema. Arch. Dis. Child..

[156] Pudney P.D.A., Mélot M., Caspers P.J., van der Pol A., Puppels G.J. (2007). An In Vivo Confocal Raman Study of the Delivery of Trans-Retinol to the Skin. Appl. Spectrosc..

[157] Pot L.M., Coenraads P.-J., Blömeke B., Puppels G.J., Caspers P.J. (2016). Real-time detection of p-phenylenediamine penetration into human skin by in vivo Raman spectroscopy. Contact Dermat..

[158] Iliopoulos F., Caspers P.J., Puppels G.J., Lane M.E. (2020). Franz Cell Diffusion Testing and Quantitative Confocal Raman Spectroscopy: In Vitro-In Vivo Correlation. Pharmaceutics.

[159] Patel A., Iliopoulos F., Caspers P.J., Puppels G.J., Lane M.E. (2021). In Vitro–In Vivo Correlation in Dermal Delivery: The Role of Excipients. Pharmaceutics.

[160] Stamatas G.N., Bensaci J., Greugny E., Kaur S., Wang H., Dizon M.V., Cork M.J., Friedman A.J., Oddos T. (2021). A Predictive Self-Organizing Multicellular Computational Model of Infant Skin Permeability to Topically Applied Substances. J. Investig. Dermatol..

